# Inflammatory biomarkers and pendelluft magnitude in ards patients transitioning from controlled to partial support ventilation

**DOI:** 10.1038/s41598-022-24412-1

**Published:** 2022-11-23

**Authors:** Rodrigo A. Cornejo, Daniel H. Arellano, Pablo Ruiz-Rudolph, Dannette V. Guiñez, Caio C. A. Morais, Abraham I. J. Gajardo, Marioli T. Lazo, Roberto E. Brito, María A. Cerda, Sedric J. González, Verónica A. Rojas, Gonzalo A. Diaz, Lorena D. M. López, Juan N. Medel, Dagoberto I. Soto, Alejandro R. Bruhn, Marcelo B. P. Amato, Nivia R. Estuardo

**Affiliations:** 1 Unidad de Pacientes Críticos, Departamento de Medicina, Hospital Clínico Universidad de Chile, Santiago, Chile; 2 Center of Acute Respiratory Critical Illness (ARCI), Santiago, Chile; 3 Departamento de Kinesiología, Facultad de Medicina, Universidad de Chile, Santiago, Chile; 4 Programa de Salud Ambiental, Facultad de Medicina, Instituto de Salud Poblacional, Universidad de Chile, Santiago, Chile; 5 Divisao de Pneumologia, Faculdade de Medicina, Instituto Do Coração, Hospital das Clinicas HCFMUSP, Universidade de São Paulo, São Paulo, Brazil; 6 Departamento de Fisioterapia, Universidade Federal de Pernambuco, Recife, Brazil; 7 Departamento de Radiología, Hospital Clínico Universidad de Chile, Santtiago, Chile; 8 Departamento de Medicina Intensiva, Facultad de Medicina, Pontificia Universidad Católica de Chile, Santiago, Chile

**Keywords:** Biophysics, Physiology, Biomarkers, Medical research, Pathogenesis

## Abstract

The transition from controlled to partial support ventilation is a challenge in acute respiratory distress syndrome (ARDS) patients due to the risks of patient-self-inflicted lung injury. The magnitude of tidal volume (V_T_) and intrapulmonary dyssynchrony (pendelluft) are suggested mechanisms of lung injury. We conducted a prospective, observational, physiological study in a tertiary academic intensive care unit. ARDS patients transitioning from controlled to partial support ventilation were included. On these, we evaluated the association between changes in inflammatory biomarkers and esophageal pressure swing (ΔP_es_), transpulmonary driving pressure (ΔP_L_), V_T_, and pendelluft. Pendelluft was defined as the percentage of the tidal volume that moves from the non-dependent to the dependent lung region during inspiration, and its frequency at different thresholds (− 15, − 20 and − 25%) was also registered. Blood concentrations of inflammatory biomarkers (IL-6, IL-8, TNF-α, ANGPT2, RAGE, IL-18, Caspase-1) were measured before (T_0_) and after 4-h (T_4_) of partial support ventilation. Pendelluft, ΔP_es_, ΔP_L_ and V_T_ were recorded. Nine out of twenty-four patients (37.5%) showed a pendelluft mean ≥ 10%. The mean values of ΔP_es_, ΔP_L_, and V_T_ were − 8.4 [− 6.7; − 10.2] cmH_2_O, 15.2 [12.3–16.5] cmH_2_O and 8.1 [7.3–8.9] m/kg PBW, respectively. Significant associations were observed between the frequency of high-magnitude pendelluft and IL-8, IL-18, and Caspase-1 changes (T_0_/T_4_ ratio). These results suggest that the frequency of high magnitude pendelluft may be a potential determinant of inflammatory response related to inspiratory efforts in ARDS patients transitioning to partial support ventilation. Future studies are needed to confirm these results.

## Introduction

The transition from controlled to partial support ventilation or spontaneous modes is necessary for withdrawing mechanical ventilation in the acute respiratory distress syndrome (ARDS). Both ventilatory strategies preserve diaphragmatic contraction and allow spontaneous breathing (SB). Likewise, SB favors less sedation as well as improvements in ventilation/perfusion matching, dorsal ventilation, gas exchange, hemodynamics, and attenuate ventilator-induced diaphragmatic dysfunction, among other beneficial effects^1–4^. However, under certain conditions, SB has been demonstrated to cause or enhance lung injury and could therefore complicate the weaning^5–8^.

Several mechanisms gathered under the name “patient self-inflicted lung injury” (or “P-SILI”) are proposed to explain how spontaneous breathing may damage the lung. They include excessive tidal volume, increased pulmonary transvascular pressure, and high esophageal pressure swings^5–9^. In addition, the existence of a pendelluft phenomenon, i.e. a shift of air from non-dependent to dependent lung regions during inspiration in supine position, may produce inhomogeneous lung inflation and regional overstretch. This phenomenon has been detected at the bedside through electrical impedance tomography (EIT) and is caused by local negative pleural pressure generated by diaphragmatic contraction not uniformly transmitted through dependent regions of the lungs^10^.

Nevertheless, there is no definitive evidence that P-SILI mechanisms such as pendelluft may effectively promote lung injury in humans. An additional challenge to advance our understanding of pendelluft is that we lack a validated approach to quantify it in magnitude and frequency, both of which may theoretically influence the impact of pendelluft on lung injury.

In the present study, we aimed to determine the potential contribution of pendelluft, esophageal pressure swing (ΔP_es_), transpulmonary driving pressure (ΔP_L_), and tidal volume (V_T_) to acute lung injury, assessed by changes in inflammatory biomarkers in plasma, during the transition from controlled to partial ventilatory support.

## Methods

### Study population

We assessed patients with moderate-severe ARDS on protective mechanical ventilation (MV) for more than 48 h, hemodynamically stable, under moderate-light sedation (Richmond Agitation-Sedation Scale (RASS) − 2 to − 3) and without paralytic agents. Patients younger than 18 years old, pregnant, with contraindications to place EIT, central nervous system injury, new sepsis or moderate-severe metabolic acidosis, were excluded.

### Study protocol

In this physiological study we prospectively monitored ARDS patients transitioning from controlled to partial support ventilation with esophageal manometry and EIT and we applied a systematic approach to measure pendelluft. In parallel, we analyzed the changes in representative biomarkers of acute lung injury to determine whether the development of pendelluft was associated with an increase in any of these biomarkers (Supplementary Figure S1).

#### Baseline respiratory mechanics and PEEP titration

Before initiating SB, respiratory system compliance was calculated by dividing V_T_ by the difference between plateau pressure and total positive end-expiratory pressure (PEEP), under volume-controlled mode with VT of 6 ml/kg of predicted body weight (PBW) and respiratory rate to keep PaCO_2_ 5.3–6.6 kPa. Optimal PEEP was defined as the PEEP associated with the lowest combination of collapse and overdistension according to EIT^11^. The patients were maintained at a semi‐recumbent position (30º to 45º) during the study protocol. Further details are provided in the supplementary file.

#### Mechanical ventilation settings

The ventilator mode was switched from volume-control ventilation (VCV) to biphasic positive airway pressure mode (BiVent, Servo-i ventilator Maquet) after detection of a regular patient’s respiratory rate (RR) (≥ 10 bpm). BiVent was applied during 4-h with a target of 10–50% of spontaneous ventilation relative to total minute ventilation (Supplementary Method S1). *Pressure-high* was adjusted for V_T_ of 6 ml/kg PBW and *Pressure-low* for optimal PEEP according to EIT; *T-high* with 0.8–1.0 s duration and *T-low* to maintain the same RR as in VCV.

#### Respiratory mechanics during SB

Airway pressure (P_aw_), esophageal pressure (P_es_), and transpulmonary pressure (P_L_) were registered synchronously with EIT monitoring using a pneumotachometer (FluxMed MBMED®). P_L_ was calculated as the difference between P_aw_ and P_es_. The correct position of the esophageal catheter (Neurovent Research Inc®, Canada) was confirmed^12^ (Supplementary Method S1).

#### Ventilatory cycle and pendelluft

**Figure 1:**
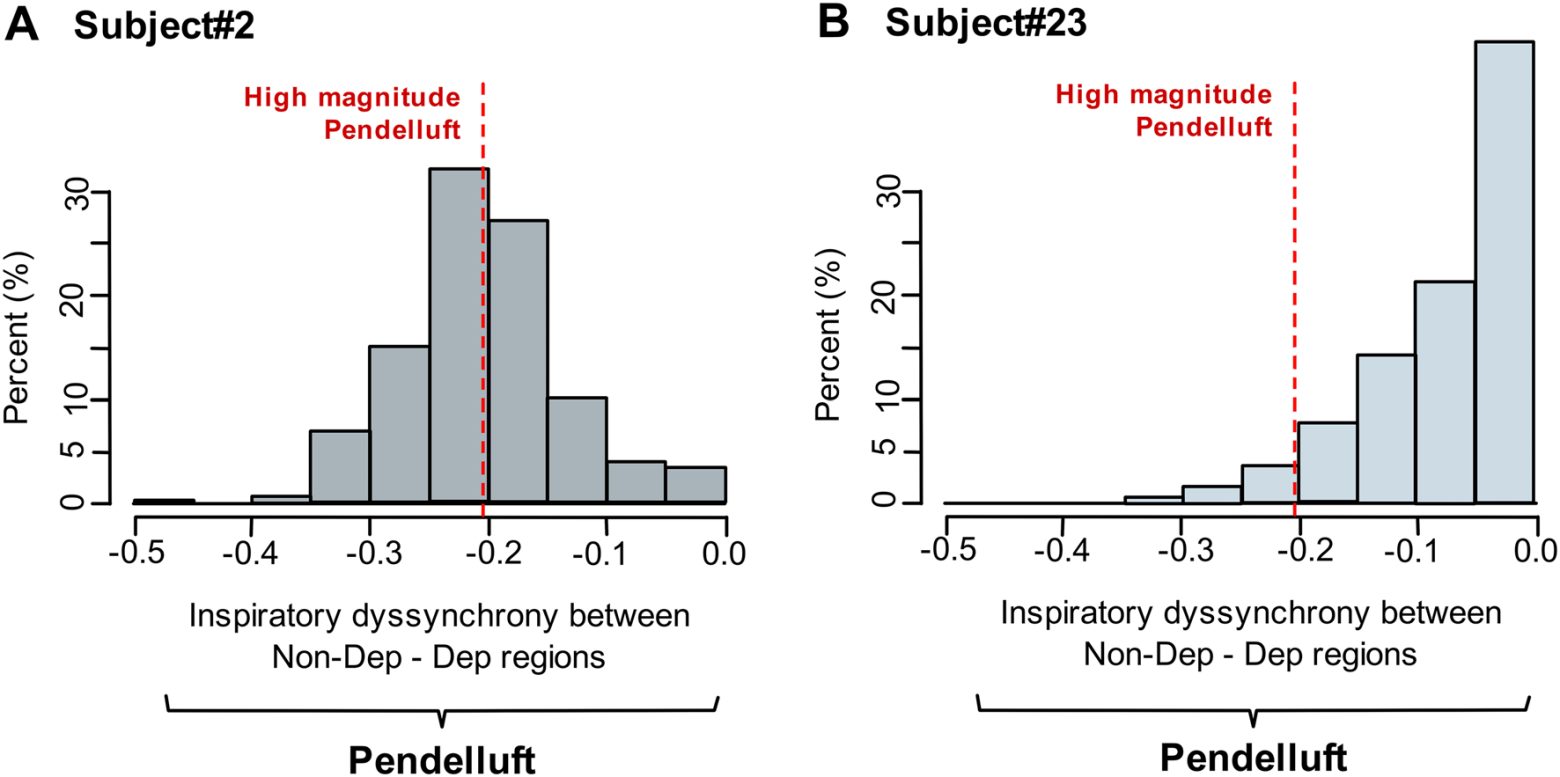
Histograms of inspiratory dyssynchrony (pendelluft) at different magnitudes. X axis corresponds to the magnitude of pendelluft and Y axis, to the percentage of ventilatory cycles with certain magnitude of pendelluft. The negative value of pendelluft represents the lost volume in non-dependent region during inspiration, expressed as fraction. The vertical red dotted line delimits the pendelluft with high magnitude cut-off − 0.2 (i.e. 20% of lost volume in non-dependent region during inspiration). (**A)** corresponds to Subject #2**,** a patient with a pendelluft mean of − 0.2 (− 20%), who presented high frequency of high-magnitude pendelluft. (**B**) corresponds to Subject #23, a patient with pendelluft mean of − 0.02 (− 0.2%), who presented low frequency of high-magnitude pendelluft.

Regional ventilation changes were analyzed in four regions-of-interest (ROI) with similar height from non-dependent to dependent regions using EIT (Enlight 1800, Timpel®, Brazil). Because controlled, spontaneous, and mixed cycles coexist in BiVent, an algorithm was implemented to define each ventilatory cycle (Supplementary Figure S2). We analyzed ventilatory cycles for the last 10-min of each monitoring hour. Pendelluft magnitude was defined as the percentage of the normalized V_T_ that moves from non-dependent to dependent regions during inspiration in each ventilatory cycle (expressed in negative values). The mean of pendelluft magnitude was obtained and the pendelluft frequency was estimated as the proportion of cycles presenting pendelluft magnitudes above specific cut-off points (− 15, − 20, − 25%, expressed as “− 0.15”, ” − 0.20″, “− 0.25″ in Fig. 1 and Supplementary Figure S3).

#### Biomarkers

Pre-specified inflammatory mediators related to acute lung injury and ventilator-induced lung injury (VILI) (pro-inflammatory cytokines [IL-6, IL-8, TNF-α], biomarkers of lung epithelial [The receptor for advanced glycation end products, RAGE] and endothelial [angiopoietin-2, ANGP2] injury, and representative biomarkers of inflammasome activation [IL-18, Caspase-1])^13–23^ were measured in serum by ELISA (Human magnetic Luminex screening assay and Human Caspase-1/ICE quantikine ELISA kit) at baseline (T_0_) and after 4 h on BiVent mode (T_4_) (Supplementary Method S3).

### Statistical Analysis

Summary statistics of pendelluft magnitude were estimated. For each patient, the pendelluft frequencies were calculated from the four 10-min monitoring periods. Friedman analysis was performed to compare changes in the pendelluft frequencies, ΔP_es_, ΔP_L_, and V_T_ during the four observation periods. The Wilcoxon signed-rank test was used to compare the biomarkers at T_0_ and at T_4_.

To evaluate the individual association between ΔP_es_, ΔP_L_, V_T_, and pendelluft frequency with each biomarker ratio [(biomarker at T_4_)/(biomarker at T_0_)], simple linear regression models were fitted. To study the independence of associations between pendelluft and ratios while controlling for ΔP_es_, ΔP_L_ and total and regional V_T_, multiple linear regressions were fitted. All the analyses were performed using R statistical software (R Foundation for Statistical Computing, Vienna, Austria).

### Ethics approval and consent to participate

This study was performed in accordance with the Declaration of Helsinki. The Institutional Review Board reviewed and approved the study (approval number N.027/2016, Comité Ético Científico Hospital Clínico Universidad de Chile). Written informed consent was obtained from all patient’s next of kin. All methods were performed in accordance with the relevant guidelines and regulation.

## Results

We included twenty-four ARDS patients of which 14 were males, with a median age of 63 [54–67] years and with body mass index of 29 [23–32] kg m^− 2^. Before enrollment their worst exchange values were median PaO_2_/FiO_2_ of 16.4 [12–20] kPa and worse sequential organ failure assessment (SOFA) score of 12 [9–14] with six patients treated with prone positioning. At the study entry, MV time was 6.5 [4–11] days, gas exchange and lung mechanics were already improving in most patients. Twelve patients presented with patchy, 4 with diffuse, and 8 with lobar computed tomography attenuations (Supplementary Table S1).

The pendelluft magnitude and its frequency for different cut-offs on hourly basis and the overall period are shown in Table 1. Nine patients (37.5%) showed a volume displacement mean ≥ 10% from non-dependent to dependent region during inspiration (i.e. mean pendelluft). Pendelluft frequency was lower at higher cut-off points of magnitude (19 [3.8–32]% at pendelluft_-15_, 10 [2–23]% at pendelluft_-20_, and 3 [1–15]% at pendelluft_-25_). ΔP_es_, ΔP_L_, and V_T_ were − 8.5 [− 10.1; − 6.6] cmH_2_O, 15.1 [12.2–16.7] cmH_2_O and 8.1 [7.2–8.8] m/kg PBW, respectively. None of these respiratory variables significantly changed during the study period (Table 1). Respiratory rate and total minute ventilation were 24 (19.6—25.9) bpm and 10.2 (8.8—12.0) L/min, respectively; both remained stable through the 4-h period (*p-*value = 0.809 and *p-*value = 0.951, respectively).

**Table 1:** Respiratory variables, mean pendelluft magnitude and pendelluft frequency at different magnitudes, during the study period.

Variable	H_1_ [Median (IQR)]	H_2_ [Median (IQR)]	H_3_ [Median (IQR)]	H_4_ [Median (IQR)]	Global [Median (IQR)]	*p-*value
ΔP_es_ (cmH_2_O)	− 7.8 (− 12.4; − 6.5)	− 9.0 (− 12.2; − 6.1)	− 8.5 (− 10.2; − 6.6)	− 8.3 (− 10.0; − 2.6)	− 8.5 (− 10.1; − 6.6)	0.905
ΔP_L_ (cmH_2_O)	15.2 (12.0–17.0)	14.8 (12.3–18.1)	14.0 (11.8–16.0)	13.7 (11.8–16.6)	15.1 (12.2–16.7)	0.714
V_T_ (mL/kg)	7.9 (6.8–8.7)	8.2 (7.0–9.6)	8.1 (7.0–8.9)	8.0 (6.9–8.8)	8.1 (7.2–8.8)	0.538
pendelluft_MEAN_ (%)	− 5 (− 11; − 3)	− 7 (− 11; − 3)	− 7 (− 12; − 2)	− 7 (− 13; − 3)	− 7 (− 11; − 3)	0.752
pendelluft_-15_ (%)	10.8 (2.7–29.3)	14.0 (2.7–32.7)	17.9 (1.6–36.7)	17.9 (4.0–39.0)	19.1 (3.8–32.2)	0.153
pendelluft_-20_ (%)	3.9 (1.1–18.7)	8.4 (0.4–21.3)	8.3 (0.6–20.3)	9.7 (1.3–24.6)	10.0 (2.1–22.7)	0.643
pendelluft_-25_ (%)	2.5 (0.0–7.2)	2.5 (0.0–9.8)	1.8 (0.2–8.2)	3.3 (0.2–10.4)	3.0 (1.2–15.2)	0.844

H_1_, H_2_, H_3_ and H_4_ represent the last 10-min of each monitoring hour within the 4-h study period; Global is the average between H_1_, H_2_, H_3_ and H_4_; pendelluft_MEAN_ represents the mean of pendelluft magnitude and pendelluft_-15_ , pendelluft_-20_ , and pendelluft_-25_, the pendelluft frequency at magnitudes − 15, − 20, and − 25%, respectively, measured in each time of monitoring; ΔP_es_: negative deflection of esophageal pressure (P_es_) from the onset of inspiratory effort during the ventilatory cycle; ΔP_L_, tidal change in transpulmonary pressure, calculated as airway pressure (P_aw)_ minus P_es_, between the maximum and minimum values of the ventilatory cycle: Both, ΔP_es_ and ΔP_L_, were measured in cmH_2_O; V_T_ (mL/kg PBW): tidal volume measured in mL/kg of predicted body weight.

**Figure 2:**
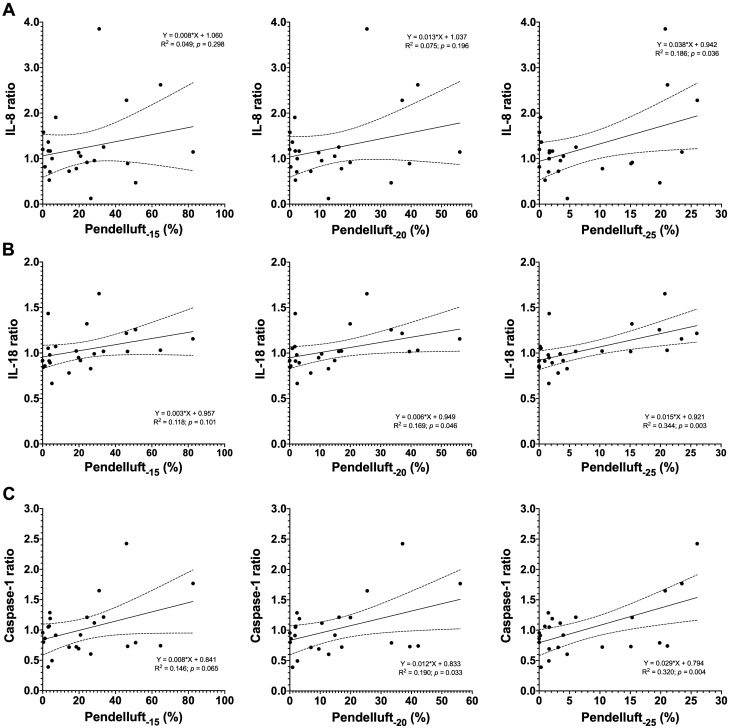
Scatter plots and regression analysis between (**A**) IL-8, (**B**) IL-18, (**C**) Caspase-1 ratios and frequencies of pendelluft magnitude − 15, − 20 and − 25%. Biomarker ratio [(biomarker at T_4_)/(biomarker at T_0_)] and the mean of pendelluft frequencies at different cut-off points of pendelluft magnitude (− 15, − 20 and − 25%), through the 4 h period of observation, were obtained for each patient. Pendelluft_-15_: mean of pendelluft frequency at magnitude of − 15%; Pendelluft_-20_: mean of pendelluft frequency at magnitude of − 20%; Pendelluft_-25_: mean pendelluft frequency at magnitude of − 25%. ΔP_es_: mean of negative deflection of esophageal pressure from the onset of inspiratory effort during the ventilatory cycle; ΔP_L_: mean of tidal change in transpulmonary pressure. V_T_: tidal volume.

**Figure 3:**
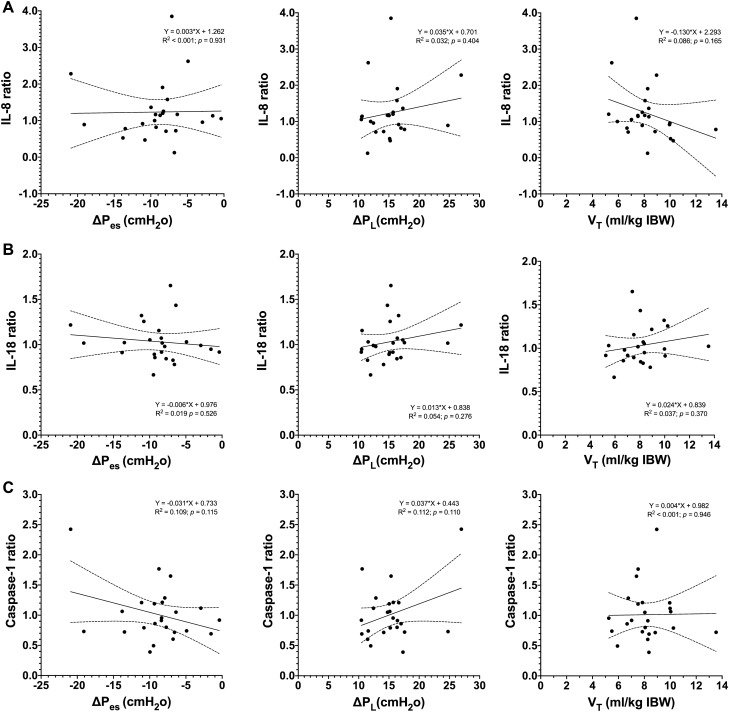
Scatter plots and regression analysis between (**A**) IL-8, (**B**) IL-18, (**C**) Caspase-1 ratios, and respiratory variables. Biomarker ratio [(biomarker at T_4_)/(biomarker at T_0_)] and the mean values by patient of ΔP_es_ , ΔP_L_ and V_T_, through the 4 h period of observation, were obtained for each patient ΔP_es_: mean of negative deflection of esophageal pressure (P_es_) from the onset of inspiratory effort during the ventilatory cycle; ΔP_L_: mean of tidal change in transpulmonary pressure. Only the association between ΔP_es_ and ΔP_L_ and the pendelluft _25_ frequency was significant (R^2^ 0.202 and *p-*value = 0.047). Several patients presented pendelluft of higher magnitude at lower than − 15 and 20 cmH_2_O of ΔP_es_ and ΔP_L_, respectively.

The overall cohort plasma concentration of the biomarkers did not change between T_0_ and T_4_ with the exception of TNF-α that decreased (Supplementary Figure S4). Nevertheless, some individual patients did exhibit an increase in biomarkers concentrations. The main results of single regression models for biomarkers ratios and mean pendelluft magnitude, frequency of different pendelluft magnitudes, or respiratory variables are shown in Fig. 2, Fig. 3 and Additional File 1 Table 2. There was only a trend in correlations between IL-18 and Caspase-1 ratios and the mean pendelluft magnitude (R^2^ 0.147 and *p-*value 0.064 for both biomarkers), but a significant association was observed between these biomarkers (and IL-8) and the frequency of high-magnitude pendelluft. The R^2^, estimate (β), and level of significance (*p-*value) increased as pendelluft magnitude became higher (from 15 to 25%) (Fig. 2). We did not observe association between other cytokines and the mean and frequency of pendelluft at any magnitude (Supplementary Table S2). Likewise, no significant associations were observed between biomarkers and global respiratory variables, with the exception of a positive correlation between RAGE ratio and ΔP_L_ (Fig. 3 and Supplementary Table S2).

**Table 2:** Effect of ΔP_es_ and ΔP_L_ on associations between pendelluft_20-25_ frequencies and biomarker ratios.

Predictor	Estimate	R^2^	t-value	*p-*value
**Model 1: IL-8 ratio = β**_**0**_** + β**_1_*pend_**20**_** + β**_2_*ΔP_**es**_				
pendelluft_-20_	1.485	0.085	1.398	0.177
ΔP_es_	0.018		0.494	0.626
**Model 2: IL-8 ratio = β**_**0**_** + β**_1_*pend_**20**_** + β**_2_*ΔP_**L**_				
pendelluft_-20_	1.324	0.075	1.286	0.212
ΔP_L_	0.002		0.056	0.956
**Model 3: IL-8 ratio = β**_**0**_** + β**_1_*pend_**25**_** + β**_2_*ΔP_**es**_				
pendelluft_-25_	4.631	0.228	2.492	0.021
ΔP_es_	0.037		1.079	0.292
**Model 4: IL-8 ratio = β**_**0**_** + β**_1_*pend_**25**_** + β**_2_*ΔP_**L**_				
pendelluft_-25_	3.942	0.188	2.200	0.039
ΔP_L_	− 0.011		− 0.266	0.793
**Model 5: IL-18 ratio = β**_**0**_** + β**_1_*pend_**20**_** + β**_2_*ΔP_**es**_				
pendelluft_-20_	0.553	0.169	1.949	0.065
ΔP_es_	− 0.001		− 0.083	0.934
**Model 6: IL-18 ratio = β**_**0**_** + β**_1_*pend_**20**_** + β**_2_*ΔP_**L**_				
pendelluft_-20_	0.558	0.169	2.038	0.054
ΔP_L_	0.001		0.086	0.932
**Model 7: IL-18 ratio = β**_**0**_** + β**_1_*pend_**25**_** + β**_2_*ΔP_**es**_				
pendelluft_-25_	1.577	0.355	3.309	0.003
ΔP_es_	0.005		0.601	0.554
**Model 8: IL-18 ratio = β**_**0**_** + β**_1_*pend_**25**_** + β**_2_*ΔP_**L**_				
pendelluft_-25_	1.500	0.348	3.327	0.003
ΔP_L_	− 0.037		− 0.370	0.715
**Model 9: Caspase-1 ratio = β**_**0**_** + β**_1_*pend_**20**_** + β**_2_*ΔP_**es**_				
pendelluft_-20_	1.025	0.235	1.856	0.078
ΔP_es_	− 0.021		− 1.112	0.279
**Model 10: Caspase-1 ratio = β**_**0**_** + β**_1_*pend_**20**_** + β**_2_*ΔP_**L**_				
pendelluft_-20_	1.141	0.225	2.131	0.045
ΔP_L_	0.021		0.978	0.339
**Model 11: Caspase-1 ratio = β**_**0**_** + β**_1_*pend_**25**_** + β**_2_*ΔP_**es**_				
pendelluft_-25_	2.611	0.336	2.659	0.001
ΔP_es_	− 0.012		− 0.646	0.525
**Model 12: Caspase-18 ratio = β**_**0**_** + β**_1_*pend_**25**_** + β**_2_*ΔP_**L**_				
pendelluft_-25_	2.728	0.334	2.952	0.008
ΔP_L_	0.012		0.654	0.520

Pendelluft_-20_ and pendelluft_-25_ represent the pendelluft frequency at magnitudes of − 20% and − 25%, respectively. ΔP_es_: negative deflection of esophageal pressure (P_es_) from the onset of inspiratory effort during the ventilatory cycle; ΔP_L_, tidal change in transpulmonary pressure, calculated as airway pressure (P_aw)_ minus P_es_, between the maximum and minimum values of the ventilatory cycle; V_T_ (mL/kg PBW): tidal volume measured in mL/kg of predicted body weight.

To explore whether pendelluft may be mediator in the pathway between global parameters and inflammatory biomarkers, simple regressions were fitted. The mean pendelluft magnitude and frequencies at different pendelluft magnitudes were associated with V_T_ at the dependent lung regions but not with total V_T_. We did not observe significant associations between pendelluft and ΔP_es_ nor ΔP_L_ suggesting that pendelluft frequency might be only partially influenced by these variables (Supplementary Table S3). Even more_,_ when ΔP_es_, ΔP_L_, and V_T_ were included as covariates in multiple linear regressions for the biomarkers ratios, the observed associations between pendelluft_20-25_ frequency and IL-8, IL-18, and Caspase-1 were only slightly attenuated, presenting stronger associations than the global parameters as evidenced by the magnitude of the t-statistic (Table 2).

## Discussion

In the present physiological study, we found that in ARDS patients transitioning from controlled to partial support ventilation: (1) the overall concentration of inflammatory biomarkers did not change consistently, although a subgroup of patients exhibited an increase in some inflammatory biomarkers; (2) the frequency of high-magnitude pendelluft was the parameter best associated with the increase in specific inflammatory biomarkers (IL-8, IL-18, and Caspase-1), independently of ΔP_es_, ΔP_L_, and V_T._

The present study describes for the first time in humans the association of high-magnitude pendelluft with the increase of specific inflammatory mediators related to VILI. The absence of a significant correlation between pendelluft and global variables, such as ΔP_es_, ΔP_L_ and V_T_ provides evidence for the current hypothesis of pendelluft as a regional phenomenon which may be related to a local change in pleural pressure^10,24,25^.

Not all patients developed high-magnitude pendelluft (Fig. 1) or increased inflammatory biomarkers, but the association between both was positive and significant. Indeed, we observed a progressive increase in R^2^, in the estimate (β), and in the level of significance (*p-*value) in relation to the increase in the cut-off points of pendelluft magnitude from 15 to 25% with the IL-8, IL-18 and Caspase-1 ratios. This suggests that the inflammatory response is triggered above a certain threshold of pendelluft in the same way as non-protective MV. High-magnitude pendelluft causes overstretch in lung regions during tidal inflation and it is known that cyclic stretch upregulates IL-8 in a strain-dependent manner^15^ and thus provides a potential explanation for the association between the frequency of high-magnitude pendelluft and the increase in IL-8. This cytokine is the major chemoattractant for neutrophils^16^ and the release of IL-8 is considered to play an important role in the inflammatory response and progression of VILI in patients with ARDS^17^.

A parallel increase in IL-18 and Caspase-1 in association with the frequency of high-magnitude pendelluft suggests a common pathway related to the inflammasome activation. IL-18 and caspase-1 have been shown to play a fundamental role in the spread of lung injury in experimental studies and in critically ill patients^18–21^. Even more, an elevated level of serum IL-18 has a suggested association with worse prognosis in patients with ARDS^18,19^. The use of MV with high V_T_ (for a few hours) has been shown to increase the expression of IL-18 and Caspase-1 in lung tissue and plasma^19^. The mechanical stress produced by MV on the lung parenchyma is capable of triggering the production of reactive oxygen species in mitochondria from activated alveolar macrophages, which activate the inflammasome leading to the processing and maturation of Pro IL-1β and Pro IL-18^20,21^.

The decision to assess inflammatory changes through the ratio of biomarker levels at T_0_ and T_4_ was based in the large inter-individual variability reported in previous studies^13–24^, as well as, the expectation that only a subgroup of patients would have intense inspiratory efforts. In addition, we believe that the biomarker ratio, by representing a relationship is sensitive to changes, discriminates the most relevant interactions and allows a more personalized analysis^26^.

Remarkably, there was a low frequency of high-magnitude pendelluft at the SB onset. Three potential causes may explain these findings: (1) In this study, we used a personalized titration of *“optimal”* PEEP according to EIT to reduce lung collapse^11^. The titrated PEEP may reduce the neuromuscular efficiency as suggested by recent physiologic studies^27,28^; (2) The ventilatory mode used (BiVent) usually generates lower tidal volumes and transpulmonary pressure than fully synchronized or partially synchronized pressure-targeted modes despite similar settings on the ventilator and patient’s effort^29^ and has shown to be a safe and potentially beneficial ventilatory strategy^30–33^. We applied a similar ventilatory strategy to that used in a randomized controlled trial with BiVent (*BiRDS study, ClinicalTrials.gov Identifier: NCT01862016*). (3) Finally, the sedation was titrated to maintain  RASS − 2 to − 3 and SB up to 50% of the total minute ventilation, which was successfully obtained and remained between 35 and 50% in all patients. In this scenario, which may be considered partially inhibitory of strong inspiratory efforts, no associations were found between the percentage of SB over total minute ventilation and pendelluft magnitude or inflammatory biomarkers.

P-SILI may be more likely when intense inspiratory efforts occur in severe ARDS ("solid-like" lungs), compared to mild ARDS ("fluid-like" lungs)^34,35^. In the present study, all patients had moderate to severe ARDS upon admission to the ICU. However, at the time of inclusion, patients had remained on MV for over a week on average and thus: gas exchange, lung disease, and lung mechanics were already improving in most patients. Nevertheless, high-magnitude pendelluft was observed in some patients despite protective values of ΔP_es_, ΔP_L_, and V_T_. Both the global parameters and pendelluft may share some common mechanisms and thus the observed associations are attenuated when including both variables in the multivariate model, but the signal from pendelluft was a stronger predictor of inflammatory response and it might be mediated by specific pathways unrelated to global parameters.

### Study Limitations

Our findings must be interpreted with caution due to several limitations such as: (1) the small sample size; (2) the use of BiVent mode with optimized PEEP and analgosedation enough to maintain spontaneous ventilation up to 50% of total minute ventilation, which may have avoided high-magnitude pendelluft and a stronger inflammatory response; (3) the short period of spontaneous ventilation; (4) the potential effect of simultaneous phenomena acting as confounders on biomarkers changes; (5) the study does not allow to infer whether the changes in IL-18, Caspase 1 and IL-8 plasma concentrations observed in patients with high-magnitude pendelluft were indeed produced in the lung; and, (7) the lack of validation of the results of pendelluft in the absence of a unified definition of the phenomenon.

For all the above, the present study should be considered as a pilot exploratory and hypothesis generating study. Further research is needed to assess the role of pendelluft in clinical practice. However, to the best of our knowledge, this is the first attempt to explore the association between pendelluft and inflammation in subjects with ARDS during the first hours of spontaneous ventilation.

## Conclusions

In conclusion, in ARDS patients transitioning from controlled to partial support ventilation high-magnitude pendelluft was independently associated with an increase in specific inflammatory biomarkers related to VILI (IL-8, IL-18 and Caspase-1). The development of pendelluft may be a potential determinant of P-SILI at the spontaneous ventilation onset. Future studies are needed to confirm this conclusion.

## Supplementary Information

Supplementary Information.

## Data Availability

All data generated or analyzed during this study are included in this published article and its supplementary information file.
