# Supplementary material for: Inflammatory biomarkers and pendelluft magnitude in ards patients transitioning from controlled to partial support ventilation
Source: Sci Rep. 2022 Nov 23;12:20233. doi: 10.1038/s41598-022-24412-1 (PMC9684576; doi:10.1038/s41598-022-24412-1)
Supplement: Supplementary file 1 — Supplementary Information. [file 41598_2022_24412_MOESM1_ESM.docx]

**Supplementary Data**

**INFLAMMATORY BIOMARKERS AND PENDELLUFT MAGNITUDE IN ARDS PATIENTS TRANSITIONING FROM CONTROLLED TO PARTIAL SUPPORT VENTILATION**

**Methods Supplement:**

- Method S1. Study Protocol
- Method S2. Definition of Pendelluft. magnitude of Pendelluft and its Frequency
- Method S3. Inflammatory Biomarkers

**Figures Supplement**

- Figure S1. Study protocol
- Figure S2. Ventilatory cycle definition
- Figure S3. Description of dyssynchrony approach for pendelluft magnitude assessment
- Figure S4. Plasma concentration of biomarkers

**Tables Supplement**

- Table S1. Demographic, clinical and radiological characteristics from patients at the study onset
- Table S2. Associations between biomarkers ratio of IL-6. TNF-α. RAGE. and ANGPT2. and the pendelluft mean. pendelluft frequency at different magnitudes or respiratory variables.
- Table S3. Associations between ΔP_es_ - ΔP_L_ - V_T_ (total tidal volume) and V_T_dep (tidal volume at dependent region) and the pendelluft mean or pendelluft frequency at different magnitudes.

**METHODS SUPPLEMENT:**

**Method S1. Study Protocol**

1. *Respiratory mechanics at baseline and PEEP titration*:

Six to twelve hours before initiating spontaneous breathing (SB) efforts. respiratory mechanics was assessed under volume-controlled ventilation mode with VT of 6 ml/kg of predicted body weight (PBW) and respiratory rate (RR) to keep PaCO_2_ 5.3-6.6 kPa. The fraction of inspired oxygen (FiO_2_) was programmed for oxygen saturation (SaO_2_) above 92% and lower than 98% (Puritan-Bennett 840 Ventilator System - Nellcor Puritan Bennett. CA). Patients at this phase were under sedation (using Fentanyl, Propofol, or Midazolam). A recruitment maneuver and decremental PEEP-titration trial were performed once insured hemodynamic stability and deep sedation (without SB efforts) were achieved (S1). Briefly, optimal PEEP was defined as the PEEP associated with the lowest combination of collapse and overdistension according to EIT monitoring. Quasistatic compliance of the respiratory system was calculated by dividing V_T_ by the difference between plateau pressure and total PEEP. Technical details were recently published by Cornejo et al. (S2).

1. *Biphasic Positive Airway Pressure mode (BiVent)*:

Patients were ventilated with a Servo-i ventilator (Maquet Critical Care. Sweden), under moderate-light sedation without neuromuscular paralysis in BiVent mode (analgesia and sedation protocol is described below). BiVent was used for a 4-hours period with a target of spontaneous ventilation between 10-50% of total minute ventilation. The ventilator’s parameters were: *Pressure-high*. adjusted for a Vt of 6ml/kg IBW; *Pressure-low.* according to optimal PEEP; *T-high* 0.8-1.0 seconds; *T-low.* adjusted for the same rate as in volume-controlled ventilation.

1. *Analgesia and sedation protocol*

This protocol provides guidance of analgosedation therapy in mechanically ventilated patients and includes diagnose tools and recommendations for medication selection.

To monitor analgesia, Behavioral Pain Scale (BPS) is applied each 2 hours, and the pharmacological therapy for this aim is Acetaminophen (in dose of 1 gram each 8 hours) plus Fentanyl (dose I.V: 0.6 - 3.6 ug/Kg/h, with an initial recommended dose of 0.6 ug/Kg/h for light sedation and 1.2 ug/Kg/h for deep sedation).

To monitor sedation, Richmond Agitation-Sedation Scale (RASS) is used. The goal of sedation therapy is defined by the medical team. Deep sedation (RASS -4 to -5) is defined for the early phase of severe ARDS. Moderate-light sedation (RASS -2 to -3) is used for the transition from controlled to partial support ventilation.

The sedative agents used are Propofol (dose IV: 0.5 – 3 mg/Kg/h), and Dexmedetomidine (dose IV: 0.2 – 1.5 ug/Kg/h, with an initial recommended dose of 0.8 ug/Kg/h I.V. in light sedation).

For the switch from deep to moderate-light sedation, the infusion dose of sedative and opioid agents was reduced in steps, initiating with a 50% decrease to ensure patient safety. The ventilator mode was switched from volume-controlled mode to BiVent after detection of a regular patient’s respiratory rate ≥10 bpm. Once the patient achieved the spontaneous breathing target, the infusion dose was maintained through the 4-hours study period and Fentanyl bolus (0.5 mcg/kg) and -exceptionally- Propofol bolus (0.25 mg/kg) were allowed. Dexmedetomidine could be used instead of Propofol following the recommendations. Paradoxical breathing, unresponsive severe agitation (RASS > +2) and hemodynamic instability during the protocol were considered as criteria of intolerance.

1. *Respiratory variables and regional ventilation monitoring*

Respiratory flow. airway pressure (P_aw_). esophageal pressure (P_es_) and transpulmonary (P_L_) were registered synchronously with EIT during ongoing mechanical ventilation using a pneumotachometer (FluxMed). and data were collected on a personal computer-based data acquisition system. An esophageal balloon (Neurovent catheter, Canada) was inserted to measure P_es_. A P-V curve was performed to understand catheter compliance of esophageal balloon “in vitro”; a non-stressed volume range was identified for use later. The catheter position was validated using a chest compression or inspiratory effort maneuver (S3). Instead of absolute values for P_es_ and P_L_, we used ΔP_es_ and ΔP_L_; both were referred from the end-expiratory level (S4). ΔP_es_ was calculated as the negative deflection of P_es_ from the onset of inspiratory effort. ΔP_L_, tidal change in transpulmonary pressure, was calculated as airway pressure (P_aw_) minus P_es_, between the maximum and minimum values during the ventilatory cycles.

Electrical impedance tomography (EIT) was continuously monitoring during BiVent to visualize the regional distribution of inflation. EIT data was recorded using the impedance tomography monitor (Enlight 1800, Timpel, São Paulo. Brazil) with 32 electrodes imbedded in a special silicon belt placed on the perimeter defining a cross sectional plane of the thorax at the level of the 5–6^th^ intercostal space. Local impedance changes were plotted in a matrix containing 860 valid pixels from a total of 1024 (32×32). Regional distribution of ventilation was analyzed with the subdivision of the thorax in four regions of interest (ROI) from non-dependent (ROI-1 and ROI-2) to dependent regions (ROI-3 and ROI-4).

**Method S2. Definition of Pendelluft. magnitude of Pendelluft and its Frequency**

Because controlled, spontaneous and mixed cycles coexist in BiVent mode, and even coincide in complex ventilatory cycles, an algorithm in R software was implemented to define each ventilatory cycle based on the EIT signals.

- For feasibility, we decided to record the last 10 minutes in every hour during this 4-hours period (represented as H_1_. H_2_. H_3_ and H_4_) to have an average representation of spontaneous breathing pattern.
- Drifts of the electrical impedance signal were smoothed in moving 30-second windows and removed from original signal so to generate a corrected signal [ROI-c]).
- Spontaneous breaths were detected with negative deflections in P_es_. Considering that changes in ROI-4 signal is closely related to the patient's inspiratory effort but also to controlled breaths, the beginning of the cycle was set at the minimum values ​​of ROI-4c. The onset of ventilatory cycle was evidenced from the slope changes in sign, from (-) to (+), of the function ROI-4c against time. To avoid intermediate undulations of the electrical impedance signal as a start of the cycle, only minimums found with a ROI-4 below a threshold were considered. The threshold extended from the lowest values of ROI-4 to a fraction of the range of ROI-4c values found, as shown in the following equation:
  - Threshold = PCTL _5_ + 0.25 * (PCTL _99_ - PCTL _5_)
- The maximum values of ROI4c were considered to define the limit of inspiration during the ventilatory cycle.
- Cycles lower than 1 second and higher than 6 seconds were excluded.
- Only cycles with inspiratory efforts were included for the analysis.

Once each ventilatory cycles were defined, pendelluft was measured in each ventilatory cycle with spontaneous breathing after phase angle visualization.

- To quantify the phenomenon, we proceeded to normalize the sum of the non-dependent regions (ROI-1 + ROI-2) and the sum of the dependent regions (ROI-3 + ROI-4). Under no pendelluft, it is expected that both signals follow a similar wave during inspiration. If pendelluft is present, ROI-1 loses volume during inspiration, producing that the wave of the non-dependent regions (ROI-1+ROI-2) would lag behind the dependent regions (ROI-3+ROI-4) evidencing the displaced volume between the regions (this was assessed observing the phase angle). Based on the above, pendelluft was quantified as the average difference between the normalized ROI-1+ROI-2 and ROI-3+ROI-4 and expressed as a percent of the normalized tidal volume. This method allows to detect and quantify pendelluft during ventilatory cycles with (Figure S3) and without inspiratory synchronization.
- Once the amount of pendelluft per cycle was defined, a pendelluft event within a cycle was determined using different magnitudes of the phenomenon (cut-off points: 15, 20, and 25% of lost volume in non-dependent region during inspiration [in negative values], are expressed as fractions -0.15, -0.20 and -0.25 in Figure 1). These thresholds are based on the correlation between the changes in ΔZ and strain (S2), knowing that strain values above ~0.15 were already enough to increase metabolic activity in the ventilated lung, or above 0.27 to promote an inflammatory response characterized by increasing biomarkers (S5, S6).
- Using the above definitions, pendelluft frequency at different magnitudes was estimated for a given time period.

This process was automated for all ventilatory cycles during EIT monitoring, being able to determine for each patient the pendelluft frequency over a certain cut-off point of magnitude (${Pendelluft}_{x} fequency$) in relation to the total number (N) of observed ventilatory cycles with spontaneous breaths:

$${Pendelluft}_{x}frequency=\frac{Number of ventilatory cycles with pendelluft>x \%}{Total number of respiratory cycles with spontaneous breaths}$$

The convenient method to assess pendelluft (*“dyssynchrony-based approach”*) differs from the recent studies detecting transiently overstretched dependent lung regions during tidal inflation, despite the use of “protective” tidal volumes (*“stretch-based approach”*) (S7). The *“stretch-based approach”* considers two spatially different lung regions, one under-stretched and another over-stretched at a certain moment during lung inflation (in inspiratory pause), detected within the same image. The *“stretch-based approach”* requires a control cycle as comparator and may be problematic when the under-stretched lung region is outside the plane of the image. The *dyssynchrony-based approach* does not require inspiratory pause nor a control cycle as comparator. This method is more sensitive to volume lost from non-dependent lung regions and maintains good correlation with regional ventilation at dependent regions, but it does not quantify the stretch in these regions. Both approaches to assess pendelluft may be considered complementary.

**Method S3. Inflammatory Biomarkers**

For biochemical analysis, prespecified cytokines in serum (IL-6, IL-8, TNF-α, RAGE, ANGPT2, IL-18 and Caspase-1) were measured with commercially available ELISA kits (Human magnetic Luminex screening assay and Human Caspase-1/ICE quantikine ELISA kit) at baseline (T_0_) and after 4 hours on BiVent mode (T_4_). Blood was collected in vials containing EDTA. Whole-blood samples were then centrifugated to separate plasma from cellular component at 3000 rpm for 10 minutes. Plasma aliquots were storage at -80 Celsius degrees until final analysis in a central batch (after patients’ recruitment and data collection had been completed). Operators in charge of handling and process the samples (biochemist head of the Laboratory D.S. and one of the research PhD student R.B.) were blind to the clinical behavior of the patients during the study period and their respiratory variables (including the offline analysis).

To evaluate the association between pendelluft mean, pendelluft frequencies at different magnitudes, or ΔP_L_, ΔP_es_ and V_T_ means, and each biomarker ratio [(biomarker at T_4_)/(biomarker at T_0_)] independently, simple linear regression models were fitted. To study the independency of the associations between pendelluft and ratios while controlling for ΔP_es_, ΔP_L_ and total and regional V_T_, multiple linear regressions were fitted.

The current manuscript is the main result of our project “Determinants of lung injury induced by spontaneous breathing efforts during the transition from controlled ventilation to partial ventilatory support in ARDS patients”, supported by the Chilean grant FONDECYT Nº 1161510, which was conducted between May 2017 and March 2020. Spontaneous breathing was targeted at 10-50% of the total minute ventilation according to the BIRDS Trial (ClinicalTrials.gov Identifier: NCT01862016). When the project was written. we did not know if breathing spontaneously at the early phase of ARDS, compared to volume assist-control ventilation, might improve the survival in moderate to severe ARDS patients, as it was going to be assessed in BIRDS Trial.

**REFERENCES:**

1. Riker RR, Picard JT, Fraser GL. Prospective evaluation of the Sedation-Agitation Scale for adult critically ill patients. *Crit Care Med* 1999; **27**:1325-9.
2. Cornejo R, Iturrieta P, Olegário TMM, et al. Estimation of changes in cyclic lung strain by electrical impedance tomography: Proof-of-concept study. *Acta Anaesthesiol Scand* 2021; **65**: 228-35.
3. Baydur A, Behrakis PK, Zin WA, Jaeger M, Milic-Emili J. A simple method for assessing the validity of the esophageal balloon technique. *Am Rev Respir Dis* 1982; **126**: 788-91.
4. Bellani G, Grasselli G, Teggia-Droghi M, et al. Do spontaneous and mechanical breathing have similar effects on average transpulmonary and alveolar pressure? A clinical crossover study. *Crit Care* 2016; **20**: 142.
5. Bellani G, Guerra L, Musch G, et al. Lung regional metabolic activity and gas volume changes induced by tidal ventilation in patients with acute lung injury. *Am J Respir Crit Care Med* 2011; **183**: 1193–9.
6. González-López A, García-Prieto E, Batalla-Solís E, et al. Lung strain and biological response in mechanically ventilated patients. *Intensive Care Med* 2012; **38**: 240–7.
7. Yoshida T, Torsani V, Gomes S, et al. Spontaneous effort causes occult pendelluft during mechanical ventilation. *Am J Respir Crit Care Med* 2013; **188**: 1420-7.

**FIGURES SUPPLEMENT**

**Figure S1. Study protocol**

Respiratory mechanics. Optimal PEEP (according electrical impedance tomography) and cyclic strain by whole-lung CTs at end-expiration and end-inspiration were assessed 6-12 hours before initiating SB efforts. Later. BiVent was used for a 4-hours period with a target of spontaneous ventilation between 10-50% of total minute ventilation (The ventilator’s parameters on BiVent were: *Pressure-high*. adjusted for a Vt of 6ml/kg IBW; *Pressure-low.* according to optimal PEEP; *T-high* 0.8-1.0 seconds; *T-low.* adjusted for the same rate as in volume-controlled ventilation). Inflammatory biomarkers in serum (IL-6. TNF-α. IFN-γ. IL-18. RAGE. ANGPT2) were measured by ELISA just before SB onset on BiVent (T_0_) and after 4 hours on BiVent mode (T_4_). Electrical impedance tomography and respiratory mechanics monitoring were registered during the study protocol. Offline analysis was performed considering different pendelluft magnitudes and frequencies.


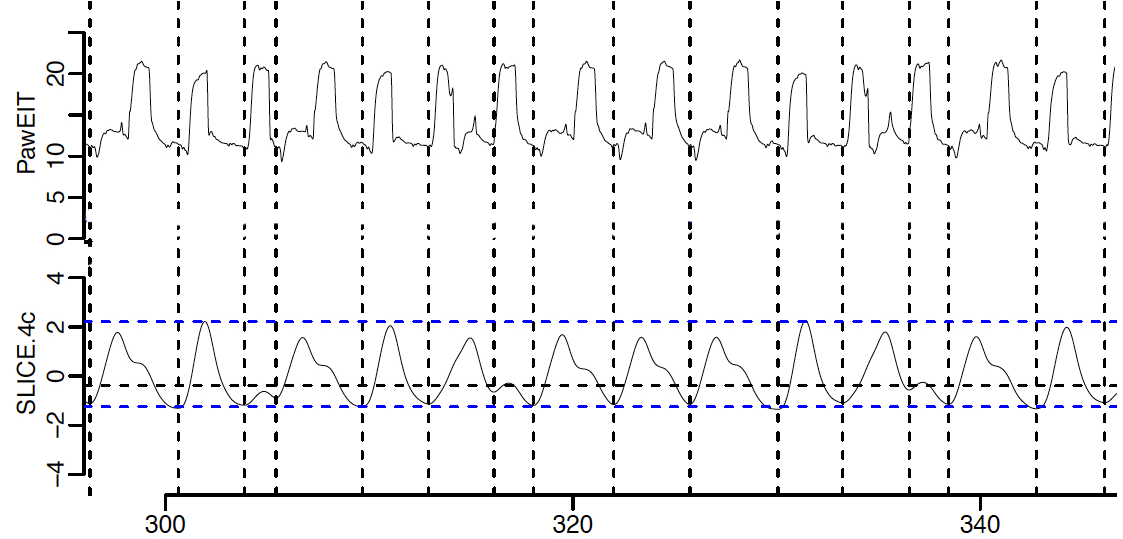


**Figure S2.** **Ventilatory cycle definition**

PawEIT: airway pressure in the EIT monitoring; SLICE 4c: corrected electrical impedance signal at SLICE 4. X axis corresponds to time in frames. Vertical lines delimit the ventilatory cycles. Cycles 3th, 7th and 13th are controlled breaths.


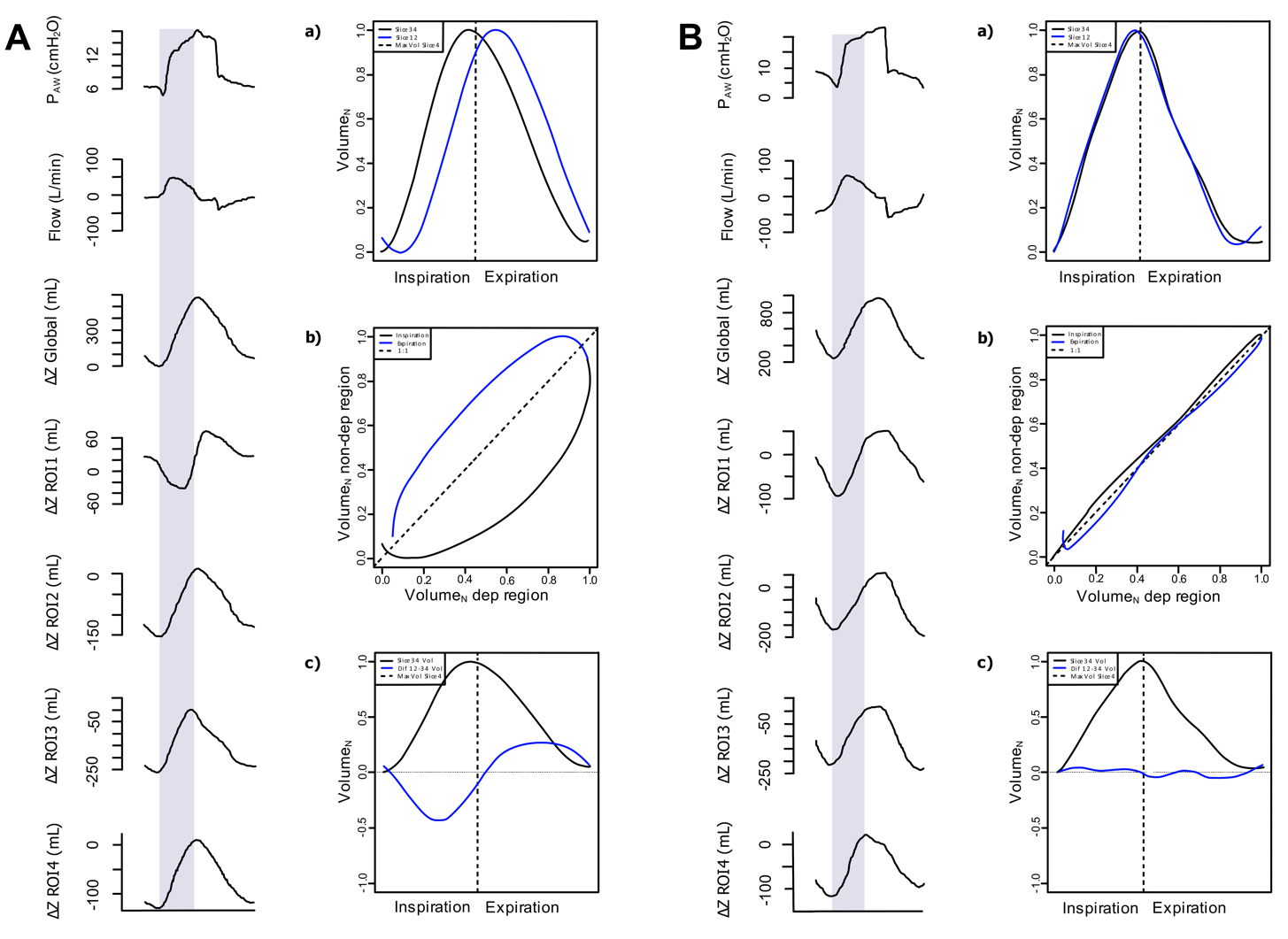


**Figure S3.** **Description of dyssynchrony approach for pendelluft magnitude assessment**

A and B illustrate the detection and magnitude assessment of pendelluft in mechanically ventilated patients with acute respiratory distress syndrome under BiVent mode and spontaneous breathing. Airway pressure (Paw) and electrical impedance tomography (EIT) signal (ΔZ) were obtained from a patient with (A) and without pendelluft (B). At left, for both A and B, EIT image was divided into four region-of-interest (ROI), each covering 25% of the ventrodorsal diameter (ROI 1–4). The dashed gray area indicates the inspiratory phase; note in A that the early inflation in the dependent region (ROI 3-4) was accompanied by concomitant (transient) deflation of non-dependent region (ROI 1), indicating volume displacement from nondependent to dependent lung regions (pendelluft phenomenon). In the other hand, in B, simultaneous inflation of each of the different lung regions was observed despite inspiratory effort detected in airway pressure (P_aw_) curve. At right, (a) dyssynchrony in lung inflation, (b) phase angle and (c) percentage of normalized volume displacement between non-dependent (ROI 1-2, in blue) and dependent regions (ROI 3-4, in black) are shown in representative cases with (A) and without (B) pendelluft. The detection process was automated for each ventilatory cycle during EIT monitoring, and through it, determining for each patient monitoring period the frequency of pendelluft over a certain cut-off point of magnitude (Pendelluft _X_ frequency) in relation to the total number (N) of observed ventilatory cycles with spontaneous breaths: Pendelluft _X_ frequency = [(Number of ventilatory cycles with pendelluft > X%) / (Total number of ventilatory cycles with spontaneous breaths)].

**
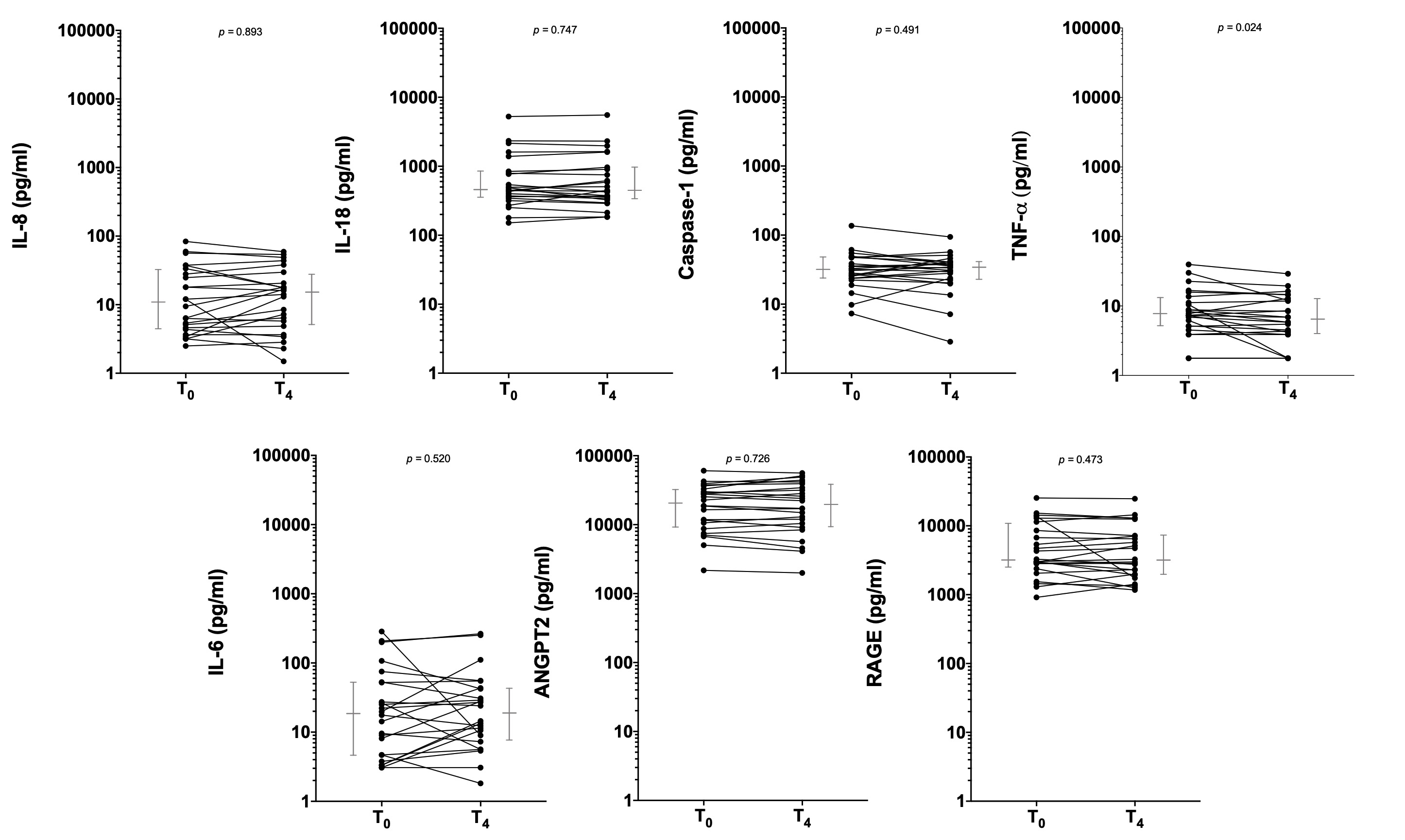
**

**Figure S4. Plasma concentration of biomarkers**

Blood samples for inflammatory biomarkers were obtained at baseline (T_0_) and after 4-hours (T_4_) on BiVent mode. This graph shows the median and interquartile ranges (25-75%) of inflammatory biomarkers at T_0_ and T_4_ (vertical parallel lines). and line plot joining the individual points defined by each patient's biomarkers concentration between T_0_ and T_4_.

**TABLES SUPPLEMENT**

**Table S1. Demographic, clinical and radiological characteristics from patients at the study onset**

| **Pt**  (N) | **Age**  (y.o) | **Sex**  (M/F) | **BMI**  (kg m^-2^) | **SOFA**  score | **CRP**  (mg/dL) | **MV**  cause | **MV time** (days) | **V_T_**  (ml/kg) | **PEEP**  (cmH_2_O) | **C_RS_** | **DP**  (cmH_2_O) | **PaO_2_/FiO_2_**  (kPa) | **CT image** |
| --- | --- | --- | --- | --- | --- | --- | --- | --- | --- | --- | --- | --- | --- |
| 1 | 51 | M | 50.8 | 2 | 69 | Bact. pneum. | 9 | 6.2 | 17 | 36.0 | 11 | 38.1 | Patchy |
| 2 | 62 | F | 27.5 | 8 | 49 | Sepsis | 7 | 6.3 | 11 | 51.4 | 7 | 30.9 | Lobar |
| 3 | 77 | M | 23.1 | 4 | 46 | Bact. pneum. | 3 | 6.1 | 10 | 47.5 | 8 | 24.7 | Patchy |
| 4 | 74 | F | 22.8 | 2 | 23 | Viral pneum. | 4 | 6.6 | 8 | 37.5 | 8 | 34.2 | Diffusse |
| 5 | 30 | M | 33.1 | 11 | 161 | OLT/SIRS | 22 | 6.5 | 10 | 30.8 | 12 | 36.6 | Patchy |
| 6 | 66 | F | 22.2 | 7 | 29 | Aspir. pneum. | 14 | 6.4 | 14 | 45.7 | 7 | 39.9 | Diffusse |
| 7 | 75 | F | 22.9 | 2 | 70 | Bact. pneum. | 16 | 6.6 | 13 | 48.7 | 9 | 38.5 | Lobar |
| 8 | 67 | M | 22.6 | 3 | 55 | Miliary TB | 22 | 5.7 | 5 | 63.3 | 9 | 37.3 | Diffusse |
| 9 | 77 | F | 35.6 | 5 | 183 | Sepsis | 9 | 6.3 | 17 | 36.7 | 9 | 29.8 | Patchy |
| 10 | 64 | F | 31.8 | 12 | 71 | Sepsis | 3 | 6.4 | 14 | 40 | 10 | 25.7 | Lobar |
| 11 | 49 | F | 20.8 | 12 | 36 | Bact. pneum. | 4 | 6.3 | 9 | 52.4 | 7 | 41.7 | Patchy |
| 12 | 64 | M | 33.6 | 10 | 208 | Sepsis | 6 | 6.4 | 11 | 53.7 | 9 | 24.7 | Lobar |
| 13 | 31 | M | 23.4 | 5 | 63 | Sepsis | 7 | 6.3 | 8 | 43.8 | 8 | 27.3 | Patchy |
| 14 | 61 | F | 31.6 | 9 | 155 | Sepsis | 11 | 6.4 | 13 | 52.0 | 8 | 43.7 | Lobar |
| 15 | 56 | F | 32.0 | 6 | 58 | Viral pneum. | 4 | 6.3 | 15 | 28.0 | 11 | 25.1 | Patchy |
| 16 | 60 | M | 35.3 | 5 | 164 | Bact. pneum. | 4 | 5.8 | 15 | 33.3 | 12 | 23.5 | Lobar |
| 17 | 63 | F | 31.1 | 7 | 36.8 | Bact. pneum.. | 4 | 6.1 | 15 | 25.9 | 13 | 44.7 | Patchy |
| 18 | 57 | M | 19 | 5 | 28.6 | Sepsis | 4 | 6.2 | 13 | 26.9 | 13 | 40.2 | Diffuse |
| 19 | 52 | M | 28.7 | 5 | 133 | Mixed pneum. | 16 | 5.8 | 7 | 23.6 | 14 | 25.1 | Patchy |
| 20 | 63 | M | 33.1 | 7 | 70 | Viral pneum. | 2 | 6.4 | 7 | 39.0 | 11 | 17.7 | Lobar |
| 21 | 73 | M | 28.4 | 5 | 140 | Bact. pneum. | 4 | 6.0 | 14 | 58.3 | 6 | 45.7 | Patchy |
| 22 | 54 | M | 30.1 | 10 | 122 | Bact. pneum. | 6 | 6.3 | 11 | 31.0 | 11 | 27.3 | Lobar |
| 23 | 32 | M | 23.4 | 13 | 15.4 | Bact. pneum. | 12 | 6.2 | 15 | 45.5 | 11 | 34.9 | Patchy |
| 24 | 67 | M | 22.0 | 15 | 258 | Aspir. pneum. | 11 | 6.4 | 10 | 28.5 | 13 | 46.5 | Patchy |

Pt: patient; M/F: BMI: body mass index; SOFA score: Sequential Organ Failure Assessment (SOFA) Score; CRP: C-reactive protein; MV: mechanical ventilation; PEEP: positive end-expiratory pressure; in this case. it corresponds to optimal PEEP adjusted according electrical impedance tomography; C_RS_: quasi-static compliance of the respiratory system; DP: driving pressure. that is. is the plateau airway pressure minus PEEP; it can also be expressed as the ratio of tidal volume to C_RS_; PaO_2_/FiO_2_: is the ratio of arterial oxygen partial pressure to fractional inspired oxygen; CT image: computed tomography findings; G-Strain: global strain; R-Strain: regional strain of dorsal region; Bact. pneum: bacterial pneumonia; Viral pneum.: viral pneumonia; Mixed pneum.: coinfection viral and bacterial. Aspir. Pneum.: aspiration pneumonia; OLT/SIRS: Orthotopic Liver Transplant/Systemic Inflammatory Response Syndrome; TB: tuberculosis

**Table S2**. Associations between biomarkers ratio of IL-6. TNF-α. RAGE. and ANGPT2. and the pendelluft mean. pendelluft frequency at different magnitudes or respiratory variables.

| **Biomarker ratio**  **(y)** | **Respiratory Variable**  **(x)** | **R^2^** | **t-value** | **p-value** |
| --- | --- | --- | --- | --- |
| **IL-6** | pendelluft_MEAN_ | 0.011 | -0.499 | 0.623 |
|  | pendelluft_-15_ | 0.007 | 0.395 | 0.697 |
|  | pendelluft_-20_ | 0.009 | 0.454 | 0.654 |
|  | pendelluft_-25_ | 0.045 | 1.020 | 0.319 |
|  | ΔP_es_ | 0.062 | -1.208 | 0.240 |
|  | ΔP_L_ | 0.023 | 0.720 | 0.479 |
|  | V_T_ | 0.002 | 0.198 | 0.845 |
| **TNF-α** | pendelluft_MEAN_ | 0.001 | -0.110 | 0.913 |
|  | pendelluft_-15_ | 0.000 | -0.024 | 0.981 |
|  | pendelluft_-20_ | 0.000 | 0.070 | 0.945 |
|  | Pendelluft_-25_ | 0.009 | 0.453 | 0.655 |
|  | ΔP_es_ | 0.001 | 0.109 | 0.914 |
|  | ΔP_L_ | 0.003 | 0.271 | 0.789 |
|  | V_T_ | 0.008 | 0.414 | 0.683 |
| **RAGE** | pendelluft_MEAN_ | 0.000 | -0.015 | 0.988 |
|  | pendelluft_-15_ | 0.000 | 0.014 | 0.989 |
|  | pendelluft_-20_ | 0.006 | 0.371 | 0.714 |
|  | pendelluft_-25_ | 0.049 | 1.066 | 0.298 |
|  | ΔP_es_ | 0.136 | -1.864 | 0.076 |
|  | ΔP_L_ | 0.165 | 2.087 | 0.049 |
|  | V_T_ | 0.072 | 1.302 | 0.206 |
| **ANGPT2** | pendelluft_MEAN_ | -0.322 | -0.322 | 0.751 |
|  | pendelluft_-15_ | 0.190 | 0.190 | 0.851 |
|  | pendelluft_-20_ | 0.366 | 0.366 | 0.718 |
|  | pendelluft_-25_ | 0.955 | 0.955 | 0.350 |
|  | ΔP_es_ | -0.624 | -0.624 | 0.539 |
|  | ΔP_L_ | 0.736 | 0.736 | 0.470 |
|  | V_T_ | 0.346 | 0.346 | 0.733 |

pendelluft_MEAN_ represents the mean of pendelluft magnitude and pendelluft_15_, pendelluft_20_, and pendelluft_25_, the pendelluft frequency at magnitudes -15, -20, and -25%, respectively. ΔP_es_: negative deflection of esophageal pressure (P_es_) from the onset of inspiratory effort during the ventilatory cycle; ΔP_L_, tidal change in transpulmonary pressure, calculated as airway pressure (P_aw)_ minus P_es_, between the maximum and minimum values of the ventilatory cycle; V_T_ (mL/kg PBW): tidal volume measured in mL/kg of predicted body weight.

**Table S3.** Associations between ΔP_es_ - ΔP_L_ - V_T_ (total tidal volume) and V_T_dep (tidal volume at dependent region) and the pendelluft mean or pendelluft frequency at different magnitudes.

| **Pend. Magnitude**  **(y)** | **Respiratory Variable**  **(x)** | **R^2^** | **t-value** | **p-value** |
| --- | --- | --- | --- | --- |
| **pendelluft_MEAN_** | **ΔP_es_** | 0.017 | 0.614 | 0.545 |
|  | **ΔP_L_** | 0.000 | 0.103 | 0.919 |
|  | **V_T_** | 0.012 | -0.513 | 0.613 |
|  | **V_T_ dep** | 0.226 | -2.535 | 0.019 |
| **pendelluft_-15_** | **ΔP_es_** | 0.021 | -0.693 | 0.496 |
|  | **ΔP_L_** | 0.000 | -0.074 | 0.941 |
|  | **V_T_** | 0.002 | 0.183 | 0.857 |
|  | **V_T_ dep** | 0.186 | 2.244 | 0.035 |
| **pendelluft_-20_** | **ΔP_es_** | 0.086 | -1.437 | 0.165 |
|  | **ΔP_L_** | 0.015 | 0.577 | 0.570 |
|  | **V_T_** | 0.004 | 0.282 | 0.781 |
|  | **V_T_ dep** | 0.200 | 2.348 | 0.028 |
| **pendelluft_-25_** | **ΔP_es_** | 0.158 | -2.030 | 0.055 |
|  | **ΔP_L_** | 0.050 | 1.073 | 0.295 |
|  | **V_T_** | 0.018 | 0.639 | 0.529 |
|  | **V_T_ dep** | 0.207 | 2.396 | 0.025 |

pendelluft_MEAN_ represents the mean of pendelluft magnitude and pendelluft_15_, pendelluft_20_, and pendelluft_25_, the pendelluft frequency at magnitudes -15, -20, and -25%, respectively. ΔP_es_: negative deflection of esophageal pressure (P_es_) from the onset of inspiratory effort during the ventilatory cycle; ΔP_L_, tidal change in transpulmonary pressure, calculated as airway pressure (P_aw)_ minus P_es_, between the maximum and minimum values of the ventilatory cycle; V_T_ (mL/kg PBW): tidal volume measured in mL/kg of predicted body weight. V_T_dep: tidal volume at dependent region, i.e. ROI3+ROI4.
